# Effect of meteorological factors on the activity of influenza in Chongqing, China, 2012–2019

**DOI:** 10.1371/journal.pone.0246023

**Published:** 2021-02-03

**Authors:** Li Qi, Tian Liu, Yuan Gao, Dechao Tian, Wenge Tang, Qin Li, Luzhao Feng, Qiyong Liu

**Affiliations:** 1 Chongqing Municipal Center for Disease Control and Prevention, Chongqing, China; 2 State Key Laboratory of Infectious Disease Prevention and Control, Collaborative Innovation Center for Diagnosis and Treatment of Infectious Diseases, National Institute for Communicable Disease Control and Prevention, Chinese Center for Disease Control and Prevention, Beijing, China; 3 Jingzhou Center for Disease Control and Prevention, Hubei, China; 4 School of Public Health (Shenzhen), Sun Yat-sen University, Guangzhou, China; 5 School of Population Medicine and Public Health, Chinese Academy of Medical Sciences & Peking Union Medical College, Beijing, China; Columbia University, UNITED STATES

## Abstract

**Background:**

The effects of multiple meteorological factors on influenza activity remain unclear in Chongqing, the largest municipality in China. We aimed to fix this gap in this study.

**Methods:**

Weekly meteorological data and influenza surveillance data in Chongqing were collected from 2012 to 2019. Distributed lag nonlinear models (DLNMs) were conducted to estimate the effects of multiple meteorological factors on influenza activity.

**Results:**

Inverted J-shaped nonlinear associations between mean temperature, absolute humidity, wind speed, sunshine and influenza activity were found. The relative risks (RRs) of influenza activity increased as weekly average mean temperature fell below 18.18°C, average absolute humidity fell below 12.66 g/m^3^, average wind speed fell below 1.55 m/s and average sunshine fell below 2.36 hours. Taking the median values as the references, lower temperature, lower absolute humidity and windless could significantly increase the risks of influenza activity and last for 4 weeks. A J-shaped nonlinear association was observed between relative humidity and influenza activity; the risk of influenza activity increased with rising relative humidity with 78.26% as the break point. Taking the median value as the reference, high relative humidity could increase the risk of influenza activity and last for 3 weeks. In addition, we found the relationship between aggregate rainfall and influenza activity could be described with a U-shaped curve. Rainfall effect has significantly higher RR than rainless effect.

**Conclusions:**

Our study shows that multiple meteorological factors have strong associations with influenza activity in Chongqing, providing evidence for developing a meteorology-based early warning system for influenza to facilitate timely response to upsurge of influenza activity.

## Background

Influenza has significant clinical and economic impacts each year. World Health Organization estimated that seasonal epidemics of influenza result in approximately 3–5 million severe illness and 290,000 to 650, 000 deaths worldwide each year [1]. China, the largest developing country in the world, has approximately 3.4 million influenza-associated outpatients and 88,100 influenza-associated excess respiratory deaths per year [2]. Understanding the epidemiology of influenza is critical for optimizing vaccination and other control measures. The influenza seasonal pattern is likely to be the outcome of complex interactions among survival and transmission of influenza virus, meteorological factors, and human behavior. Among which meteorological factors appear to be one of the most important. The association between weather conditions and influenza activity varied across regions and the transmission patterns of seasonal influenza were diverse even in neighboring regions sharing similar climate [3]. Therefore, it is critical to specially assess the response of influenza to meteorological factors on the local basis.

With the latitude of 29.6°N and a subtropical climate with four distinct seasons, Chongqing is the largest municipality with over 30 million registered inhabitants in China. Our previous studies demonstrated a substantial influenza mortality burden in Chongqing [4], and absolute humidity has a significant impact on influenza and pneumonia mortality among elderly people [5]. However, it remains unclear whether other meteorological factors such as temperature, relative humidity, precipitation, wind speed and sunshine affect the activity of influenza. In this study, we aimed at examining the relationships between multiple meteorological factors and influenza activity in Chongqing. This result will contribute to a better understanding of the health impacts of meteorological factors on influenza and provide more evidence for developing public health strategies and measures to reduce the high burden of influenza in Chongqing.

## Materials and methods

### Study area

This study is conducted in Chongqing, which covers an area of 82,400 km^2^ in Southwestern China and has approximately 33 million registered residents in 2019. It has a subtropical humid monsoon climate, a long and hot summer, and a short and warm winter.

### Influenza surveillance data

The influenza-like-illness (ILI) was defined as patient who has acute respiratory infection with fever and at least one respiratory symptom (cough and/or sore throat). ILIs and influenza virus positive rates were obtained from sentinel influenza surveillance network in Chongqing, which have been stated in a previous study [6]. Briefly, seven sentinel hospitals were selected based on higher accessibility to patients, higher qualifications of medical staff, adequate specimen storage capacity, and the desire of the physicians and nurses to participate voluntarily in the surveillance program. At each sentinel hospital, trained nurses and clinicians collected data on the counts of visits and the total number of ILI to outpatient and/or emergency departments, and collected nasopharyngeal swabs specimens then tested influenza virus by reverse transcription-polymerase chain reaction.

In this study, the activity of influenza virus was represented by weekly confirmed influenza cases every ten thousand outpatient visits, which was calculated by multiplying the weekly positive rates of influenza by the weekly ILI counts, on the scale of every 10,000 of the outpatient visits, similar to a previous study [7].

### Meteorological data

We obtained simultaneous weekly meteorological data, including mean temperature (°C), relative humidity (%), wind speed (m/s), sunshine (hours), as well as aggregate rainfall (mm), observed in 12 weather monitoring stations in Chongqing from the China Meteorological Data Sharing Service System (http://data.cma.cn/). We calculated averages for each meteorological variable to represent the whole Chongqing level, and no data were missing during the study period.

Absolute humidity (AH) was defined as the weight of water vapor per unit volume of air expressed as g/m^3^. We calculated AH by using temperature (°C) and relative humidity (%) obtained from China Meteorological Data Sharing Service System following the formula:

$$AH(g/m^{3})=\frac{6.112\times e^{\left[(17.67\times T)/(T+243.5)\right]}\times RH\times 2.1674}{273.15+T}$$

### Statistical analysis

Since the relationships between meteorological factors and influenza activity in the population are nonlinear and always lasting well beyond the exposure period, we used distributed lag nonlinear models (DLNMs) to assess the associations between meteorological factors and influenza activity. The DLNM proposed by Gasparrini is a flexible model that estimated the nonlinearity and distributed lag effects of exposure-response relationships simultaneously [8], especially the effects of meteorological factors on health.

We established DLNMs for mean temperature, absolute humidity, relative humidity, aggregate rainfall, wind speed and sunshine, respectively. The model for each climate variable was adjusted with other explanatory variables of meteorological factors, seasonality and long-term trend, and school holiday. Consistent with previous studies, we used the variance inflation factor (VIF) to assess the co-linearity. A VIF greater than 5 indicates multicollinearity [9]. Moreover, meteorological variables with high correlation were not combined in a model.

In order to avoid over-dispersion of the influenza activity, a Poisson regression was constructed with a quasi-Poisson function that allows for over-dispersion in the weekly influenza cases to combine DLNMs. The model structure is stated as following:

$$\log[E\left(Y_{t}\right)]=a+cb\left(climate\;variables,\;lag,\;df\right)+\sum ns\left(X_{j},\;df\right)+ns\left(time,\;df*8\right)+factor\left(holiday\right)$$

Where *E(Yt)* is t expected weekly confirmed influenza cases every ten thousand outpatient visits on week t; *α* is the intercept; *cb()* represents the cross-basis matrix of climate factor; *ns*(.) is a cubic spline function; holiday is an indicator variable which equals to 1 if week t is in school holidays and 0 otherwise. Time refers to seasonality and long-term trends in influenza, which is controlled using a cubic spline function with 5 degrees of freedom (*df*) per year; *X*_*j*_ refer to other explanatory variables of meteorological factors, except for the climate factor in cross-basis matrix.

The model was established for each meteorological factor (mean temperature, absolute humidity, relative humidity, aggregate rainfall, wind speed and sunshine), respectively. In each model, we used Akaike Information criterion (AIC) to choose the dfs for climate factor and *Xj*, which was supported by other references [10–13]. The smallest AIC value demonstrates the preferred model. The weeks of lag structure in the models were determined by the AIC, incubation period and infectious period of influenza virus [14], and other references [10, 11], which were defined between two weeks and four weeks. We used a natural cubic B-spline with two internal knots spaced at equally spaced in the log scale. We provided all AIC values and models established in this study in the S1 File.

We calculated the relative risk (RR) with corresponding 95% confidence interval (CI), relative to the reference levels. The reference levels were defined as the median values of mean temperature, absolute humidity, relative humidity, wind speed, sunshine and aggregate rainfall. Moreover, we estimated the extreme effects by comparing the 97.5^th^ percentiles and 2.5^th^ percentiles to the median values.

### Sensitivity analysis

To test the robustness of our results, sensitivity analyses were performed by using 4–7 df for time trends, 2–5 df for climate variables and other explanatory variables of meteorological factors, 2–4 maximum lag weeks, and changing the location of knots for lag-response in the model.

All statistical tests were two-sided, the *p*-value <0.05 was considered statistically significant. We performed all data analyses using R software version 3.4.2 and used the “dlnm” package for the DLNMs.

## Results

### General characteristics

**Fig 1:**
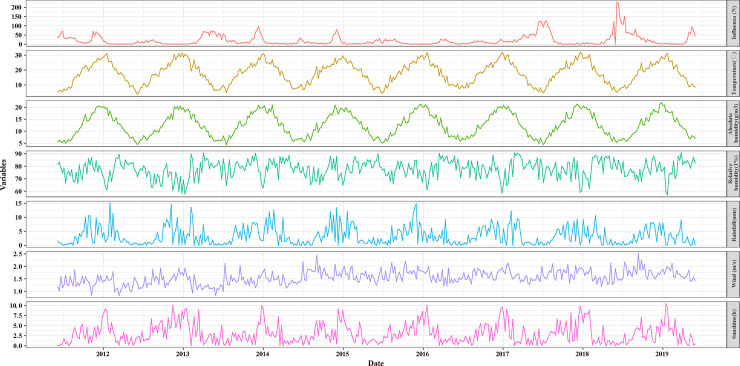
The distribution of weekly confirmed influenza cases every ten thousand outpatient visits and meteorological variables in Chongqing, China, 2012–2019.

From January 1, 2012 to December 31, 2019, a total of 43664 specimens were tested in network laboratories and 18.16% (7928/43664) were positive for influenza virus. Fig 1 displayed the time-series distribution of the weekly meteorological factors and confirmed influenza cases every ten thousand outpatient visits during the study period. From top to bottom, the panels represent the distribution of weekly confirmed influenza cases every ten thousand outpatient visits, temperature, absolute humidity, relative humidity, aggregate rainfall, wind speed and sunshine. We observed a significant seasonal variation for both influenza and meteorological factors. The weekly average mean temperature, average absolute humidity, average relative humidity, aggregate rainfall, average wind speed, and average sunshine were provided in Table 1.

**Table 1:** Descriptive statistics of meteorological factors and influenza activity in Chongqing, China, 2012–2019.

	Mean	SD	Min	P25	P50	P75	Max
Influenza	20.61	30.13	0.00	2.00	9.00	26.00	228.00
Tmean (°C)	17.88	7.49	3.55	10.57	18.18	24.18	31.85
AHmean(g/m^3^)	12.66	4.96	4.34	7.96	12.49	17.21	21.94
RHmean (%)	77.59	7.06	57.07	73.65	78.26	82.83	90.64
Rainfall (mm)	3.34	3.27	0.00	0.72	2.22	5.11	15.02
WSmean (m/s)	1.56	0.27	0.83	1.38	1.55	1.75	2.52
SUNmean (hour)	3.04	2.47	0.00	1.01	2.36	4.48	10.43

SD: standard deviation; Max: maximum; Min: minimum; Influenza: confirmed influenza cases every ten thousand outpatient visits; Tmean: mean temperature; AHmean: mean absolute humidity; RHmean: mean relative humidity; Rainfall: aggregate rainfall; WSmean: mean wind speed; SUNmean: mean sunshine. P25: the 25^th^ percentile; P50: the 50^th^ percentile; P75: the 75^th^ percentile.

Spearman correlations between weekly meteorological variables and influenza activities were showed in Table 2.

**Table 2:** Spearman's correlation results between weekly meteorological variables and confirmed influenza cases every ten thousand outpatient visits in Chongqing, China, 2012–2019.

	Influenza	Tmean	AHmean	RHmean	Rainfall	WSmean	SUNmean
Influenza	1.000						
Tmean	-0.409**	1.000					
AHmean	-0.395**	0.979**	1.000				
RHmean	0.072	-0.282**	-0.108*	1.000			
Rainfall	-0.295**	0.544**	0.620**	0.257**	1.000		
WSmean	-0.126*	0.360**	0.302**	-0.408**	0.235**	1.000	
SUNmean	-0.254**	0.684**	0.572**	-0.710**	0.121*	0.351**	1.000

*P<0.05

**P<0.01; Tmean: mean temperature; AHmean: mean absolute humidity; RHmean: mean relative humidity; Rainfall: aggregate rainfall; WSmean: mean wind speed; SUNmean: mean sunshine.

### Associations between meteorological variables and influenza activity

**Fig 2:**
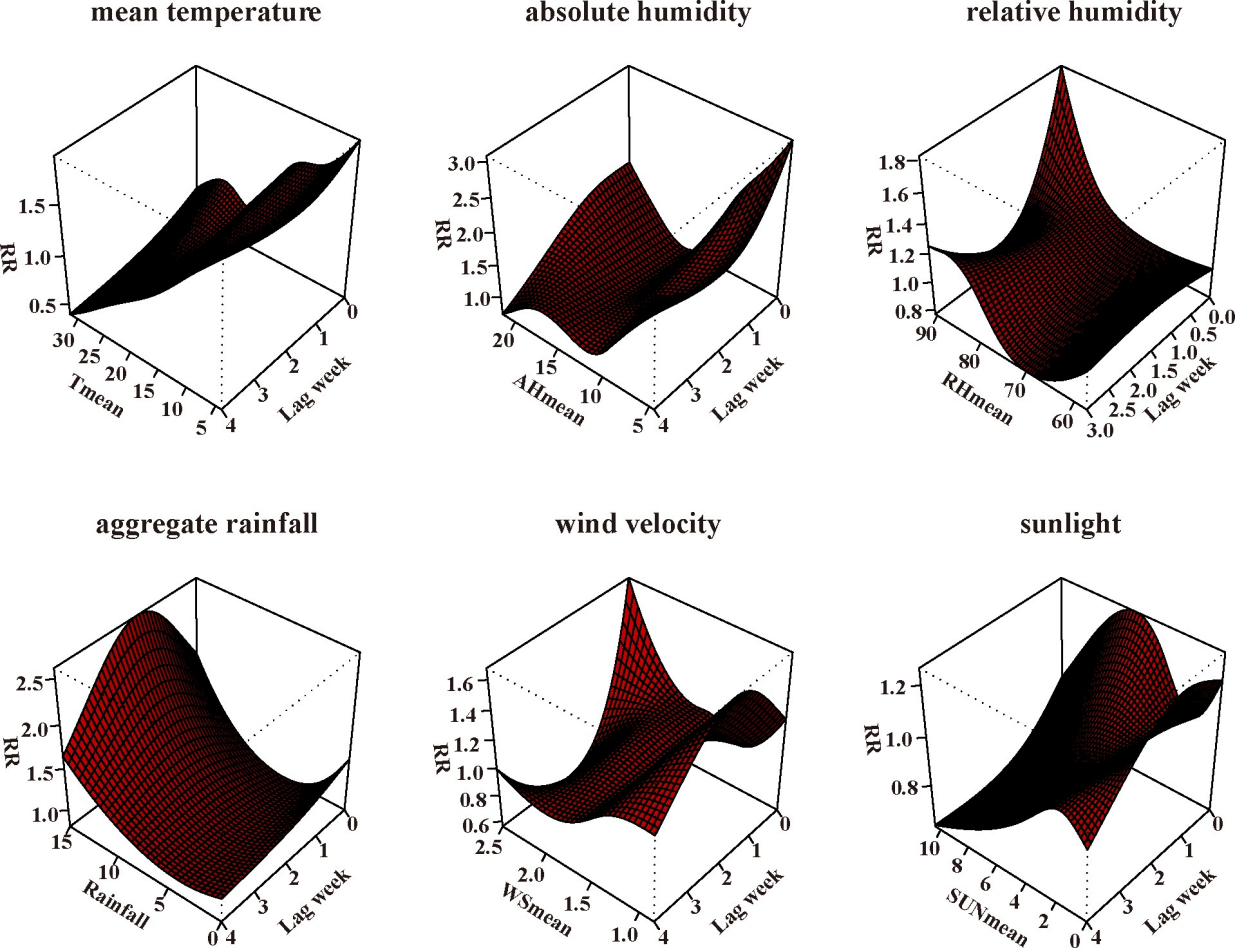
Plot of the relative risks of meteorological factors on influenza activity in Chongqing, China, 2012–2019.

**Fig 3:**
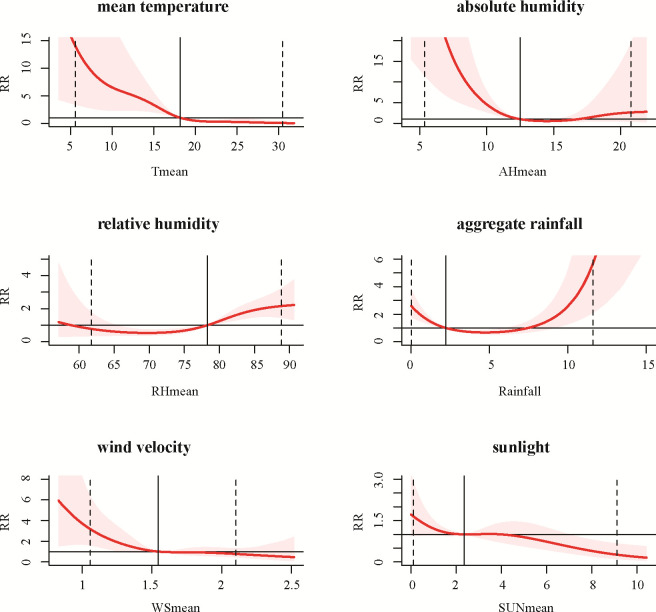
The estimated overall effects of mean temperature, absolute humidity, relative humidity, aggregate rainfall, wind speed, and sunshine along corresponding lag days. In each panel, the y-axis represents the value of relative risk, and the x-axis represents the values of the corresponding relevant variable. The red line and grey region represent the relative risk and its 95% confidence interval, respectively. The black vertical line represents the median of the corresponding meteorological factor, and the two dashed lines represent the 2.5 percentile and the 97.5 percentile for the corresponding meteorological factor, respectively.

The three-dimensional plots in Fig 2 showed the relationship between the meteorological variables and influenza activity in Chongqing with various lag weeks. For a better interpretation, the relative risks (RRs) and 95% CIs of influenza were plotted against the risk at the reference levels for mean temperature, absolute humidity, relative humidity, wind speed, sunshine and aggregate rainfall over the corresponding lag weeks in Fig 3. In general, multiple meteorological factors were associated with influenza activity. The RRs increased as weekly average mean temperature fell below 18.18°C, average mean absolute humidity fell below 12.66g/m^3^, average wind speed fell below 1.55 m/s and average sunshine fell below 2.36 hours. The relationship between weekly aggregate rainfall and influenza activity could be described as a U-shaped curve. The RRs increased as aggregate rainfall was below 2.22 mm or above 7.47 mm per week. The risk of influenza activity increased with rising average relative humidity with 78.26% as the break point. More details were provided in Fig 3 and Table 3.

**Table 3:** The highest RRs for influenza activity and corresponding meteorological factors in Chongqing, China, 2012–2019.

Climate variables	Peak 1		Peak 2	
	Value	RR (95%CI)	Value	RR (95%CI)
Tmean	3.55°C	**22.02, 95%CI: 4.28–113.39**	NA	NA
AHmean	4.34g/m^3^	**71.76, 95%CI: 15.52–331.75**	NA	**NA**
RHmean	57.06%	1.19, 95%CI: 0.29–4.77	90.63%	**2.22, 95%CI: 1.31–3.77**
Rainfall	0mm	**2.61, 95%CI: 1.76–3.87**	15.02mm	**40.37, 95%CI: 7.13–228.56**
WSmean	0.83m/s	**5.93, 95%CI: 1.51–23.22**	NA	NA
SUNmean	0	1.72, 95%CI: 0.93–3.17	NA	NA

RR: relative risk; Tmean: mean temperature; AHmean: mean absolute humidity; RHmean: mean relative humidity; Rainfall: aggregate rainfall; WSmean: mean wind speed; SUNmean: mean sunshine. Bold numbers indicate effects of meteorological factors on influenza activity are significant on the 95% confidence limit.

**Fig 4:**
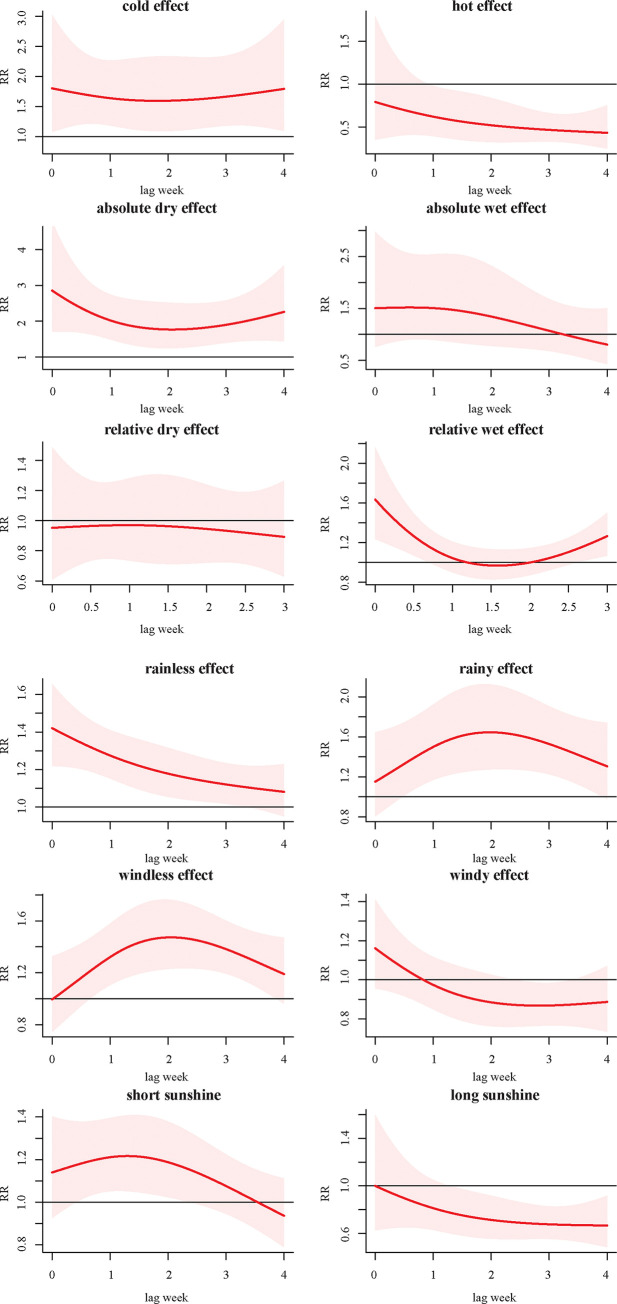
A. The extreme effects of mean temperature, absolute humidity and relative humidity with extreme high effects (97.5%) and extreme low effects (2.5%) at corresponding lag weeks. In each panel, the y-axis represents the value of relative risk, and the x-axis represents the value of lag week. The red line represents mean relative risk and grey region represent 95% confidence interval. B. The extreme effects of aggregate rainfall, wind speed and sunshine duration with extreme high effects (97.5%) and extreme low effects (2.5%) at corresponding lag weeks. In each panel, the y-axis represents the value of relative risk, and the x-axis represents the value of lag week. The red line represents mean relative risk and grey region represent 95% confidence interval.

To identify the extreme effects, the estimated effects of mean temperature, absolute humidity, relative humidity, wind speed, sunshine and aggregate rainfall comparing the 95^th^ percentiles to the median values and 5^th^ percentiles to the median values were plotted in Fig 4A and 4B. A significant cold effect was observed along 0–4 lag weeks, and hot effect appeared within 1–4 lag weeks. The absolute dry effect was observed along 0–4 lag weeks. The relative dry effect was not significant, whereas relative wet effect was observed at the current week. The rainless effect appeared within 0–3 lag weeks, and extreme rainfall effect was observed within 0.5–4 lag weeks. The windless effect was observed within 0.5–4 lag weeks, and windy effect was not significant. The short-sunshine effect was observed within 0.5–2 lag weeks, and long-sunshine effect was appeared with 1.5–4 lag weeks.

Sensitivity analyses were performed to check the robustness of our results. The residuals of the model for influenza were randomized distributed and independent over time (S2 File). We changed the maximum lag weeks, the df for climatic variables, other explanatory variables of meteorological factors and long-term trend, and the knots for lag-response, similar effects of meteorological factors on influenza activity were observed, indicating the robustness of our results (S3 and S4 Files).

## Discussion

Our study comprehensively explored the role of multiple meteorological factors on influenza activity in the largest municipality in China. The correlations between mean temperature, absolute humidity, wind speed, sunshine and influenza activity were illustrated with inverted J-shaped curves. The relation between relative humidity and influenza activity was described as a J-shaped curve. The relationship between aggregate rainfall and influenza activity was illustrated with a U-shaped curve.

Consistent with previous studies [10–12, 15, 16], we found that the mean temperature was inversely associated with influenza activity. The influenza activity increased significantly with a lower temperature below 18°C. Laboratory studies showed that low temperature may promote the spread of influenza by lengthening the survival of influenza virus, enhancing the transmissibility of influenza virus, and increasing the host susceptibility [17, 18]. Moreover, people are likely to spend more time indoor under cold condition, so the indoor environment-virus-host interactions substantially increase the opportunity of influenza transmission [7, 19].

Relative humidity is a function of water vapor and temperature, while absolute humidity is defined as absolute mass of water in the air per unit volume. Previous studies have explored the role of both absolute humidity and relative humidity on influenza survival and transmission, concluding that absolute humidity had better control on influenza survival and transmission than relative humidity [18, 20–22]. In this study, we found that mean absolute humidity was inversely associated with influenza activity, while a positive relation was observed between extreme high relative humidity and influenza. Till now, the mechanism behind the association between humidity and influenza is still lacking full understanding. Laboratory studies in guinea pigs models have indicated that low absolute humidity levels could facilitate the survival and transmission of influenza virus [17, 18]. Influenza virus could attack the innate defense of host nasal epithelia, and it was more productive and transmittable in dry weather conditions [23]. Another study [24] indicated that at high relative humidity (i.e. 99%~100% or physical conditions), viruses tend to stay stable as physiological salt concentrations are maintained, and at the lowest relative humidity (<50%) salts content of virus droplets get crystallized and the stability of viruses is maintained. The lack of association between the “relative dry”condition with influenza activity in our study may be partly explained by the fact that no exposure to extremely dry condition of relative humidity below 50% was observed during the whole study period. The relative humidity was high all year around in Chongqing, with minimum weekly average relative humidity of 57.07% during 2012–2019.

Previous studies have inconsistent findings on the association between rainfall and influenza. Many studies reported increased influenza circulation during the rainy seasons [25, 26], while others reported no or contradicting effects of rainfall [11, 27]. The findings of our study agree with the former. We found that extreme rainfall increased the risk of influenza activity with high relative risk of 40.37, which was much higher than rainless effect (relative risk was 2.61). Rainfall may lead to indoor crowding and consequently increase the probability for close contact which could speed the transmission of influenza virus [28]. Previous study also indicated that low level precipitation could increase the amount of virus particulate in the air and increased the risk of virus infection [29]. In the future, more studies are needed to fully solve the inconsistence in the association between rainfall and influenza.

The understanding of effects of sunshine and wind speed on influenza is still limited. We found that long sunshine decreases the risk of influenza activity. It has been proposed that sunshine could affect the influenza activity through the mediation effect of Vitamin D synthesis on individuals’ immune response to infection [30, 31], but remains unverified. In addition, our study showed that low wind speed increases the risk of influenza activity in Chongqing, which was consistent with previous study [32]. Future studies are needed to fully understand the roles of these meteorological factors on influenza activity and the potential mechanisms.

This study has several limitations. First, the meteorological data were taken from fixed monitoring sites rather than individual exposure measures, which may create measurement errors in the exposure. However, these errors are likely to be random. Second, due to data unavailability, we did not account for the effect of other factors such as socioeconomic condition, host susceptibility and vaccination status on the association between meteorological factors and influenza activity. Third, we didn’t explore the effects of age and sex on the associations between meteorological factors and influenza activity. We plan to address this limitation in future work. Finally, we didn’t explore the interactions among meteorological factors on influenza in this study. Further studies are needed to explore the interactions.

## Conclusions

Our study provides evidence that multiple meteorological factors have strong associations with influenza activity in Chongqing. Accordingly, a meteorology-based early warning system for influenza should be developed and implemented to facilitate timely response to upsurge of influenza activity.

## Supporting information

S1 File The Akaike Information Criterion values by changing the maximum lag weeks and the degrees of freedom for meteorological factors, and the distributed lag nonlinear models established in this study. (DOCX)

S2 File The scatter plots of residuals over time for models in this study. (TIF)

S3 File Sensitivity analyses by changing the maximum lag periods (2–4 weeks), the degrees of freedom (2–5) for climatic variables, the degrees of freedom (4–7 per year) for long-term trend, and the knots for exposure-response. Each black line represents a combination and the red line stands the model established in this study.
(TIF)

S4 File Sensitivity analyses by changing the degrees of freedom (2–4) for explanatory variables of meteorological factors. Each black line represents a combination and the red line stands the model established in this study.
(TIF)

S5 File Relevant data of this study. (XLSX)
