# Supplementary material for: Effect of meteorological factors on the activity of influenza in Chongqing, China, 2012–2019
Source: PLoS One. 2021 Feb 3;16(2):e0246023. doi: 10.1371/journal.pone.0246023 (PMC7857549; doi:10.1371/journal.pone.0246023)
Supplement: S1 File — (DOCX) [file pone.0246023.s001.docx]

**S1 File.**

**Table S1.** The Akaike Information Criterion (AIC) values by changing the maximum lag weeks and degrees of freedom (df) for meteorological factors.

| Variables | Maximum lag (weeks) | df | | | |
| --- | --- | --- | --- | --- | --- |
|  |  | 2 | 3 | 4 | 5 |
| Tmean | 2 | 3641.29 | 3634.53 | 3640.98 | 3543.80 |
|  | 3 | 3516.20 | 3517.76 | 3538.42 | 3480.77 |
|  | 4 | 3429.69 | 3433.03 | 3452.12 | **3418.19** |
|  | 2 | 3678.26 | 3528.87 | 3568.48 | 3537.58 |
| AHmean | 3 | 3677.17 | 3514.09 | 3555.54 | 3500.89 |
|  | 4 | 3577.29 | 3374.18 | 3399.51 | **3328.70** |
| RHmean | 2 | 3693.73 | 3722.02 | 3741.75 | 3756.64 |
|  | 3 | 3573.13 | **3562.2** | 3578.53 | 3598.66 |
|  | 4 | 3603.87 | 3573.86 | 3593.15 | 3597.1 |
| Rainfall | 2 | 3675.11 | 3698.1 | 3653.63 | 3687.18 |
|  | 3 | 3643.41 | 3673.73 | 3635.17 | 3675.36 |
|  | 4 | **3607.6** | 3646.25 | 3612.2 | 3646.78 |
| WSmean | 2 | 3717.78 | 3722.88 | 3732.06 | 3759.68 |
|  | 3 | 3585.86 | 3590.57 | 3581.05 | 3614.36 |
|  | 4 | 3564.46 | 3564.13 | **3554.77** | 3583.77 |
| SUNmean | 2 | 3688.92 | 3629.61 | 3648.39 | 3676.17 |
|  | 3 | 3570.61 | 3504.52 | 3510.14 | 3523.76 |
|  | 4 | 3536.93 | **3492.5** | 3497.76 | 3496.76 |

Tmean: mean temperature; AHmean: mean absolute humidity; RHmean: mean relative humidity; Rainfall: aggregate rainfall; WSmean: mean wind speed; SUNmean: mean sunshine. Bold numbers indicate the minimum AICs.

**Models established in this study**

Tmean:

glm(Influenza~cb1.temp+ns(number,df1)+ns(RHmean,3)+ns(precipitation,3)+ns(Spmean,3)+ ns(sunligh,3)+holiday, family=quasipoisson, data=Mydata)

AHmean: glm(Influenza~cb1.ah+ns(number,df2)+ns(RHmean,3)+ns(precipitation,3)+ns(Spmean,3) + ns(sunligh,3)+holiday, family=quasipoisson, data=Mydata)

RHmean:

glm(Influenza~cb1.rh+ns(number,df3)+ns(Tmean,3)+ns(precipitation,3)+ns(Spmean,3) + ns(sunligh,3)+holiday, family=quasipoisson, data=Mydata)

Precipitation:

glm(Influenza~cb1.precip+ns(number,df4)+ns(Tmean,3)+ns(RHmean,3)+ns(Spmean,3) + ns(sunligh,3)+holiday, family=quasipoisson, data=Mydata)

Spmean:

glm(Influenza~cb1.sp+ns(number,df5)+ns(Tmean,3)+ns(precipitation,3)+ns(RHmean,3) + ns(sunlight,3) +holiday, family=quasipoisson, data=Mydata)

Sunlight: glm(Influenza~cb1.sun+ns(number,df6)+ns(Tmean,3)+ns(precipitation,3)+ns(RHmean,3)+ns(Spmean,3)+holiday, family=quasipoisson, data=Mydata)
