# Supplementary figures and images for: Effect of meteorological factors on the activity of influenza in Chongqing, China, 2012–2019
Source: PLoS One. 2021 Feb 3;16(2):e0246023. doi: 10.1371/journal.pone.0246023 (PMC7857549; doi:10.1371/journal.pone.0246023)

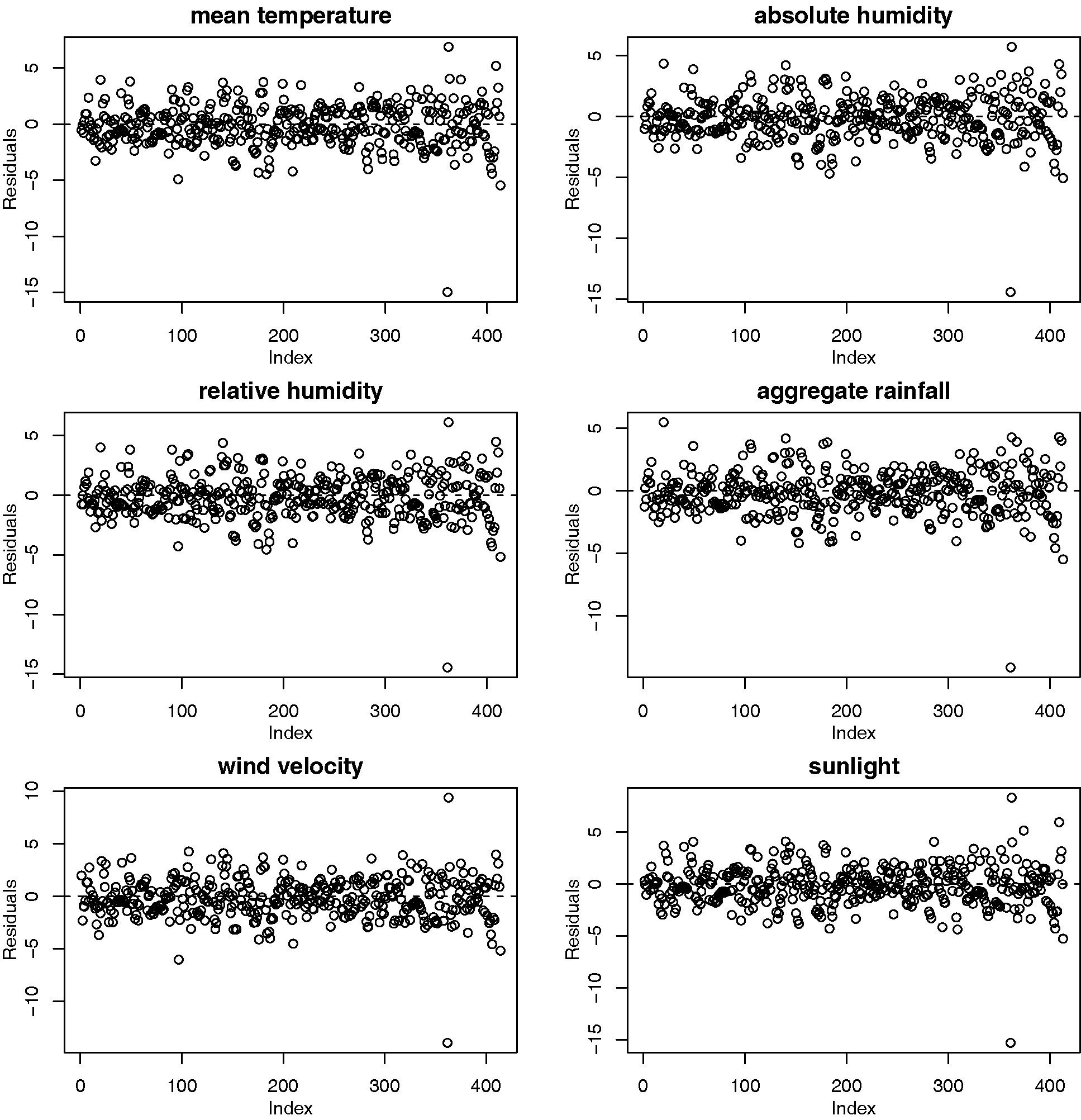

Supplement: S2 File — (TIF) [file pone.0246023.s002.tif]

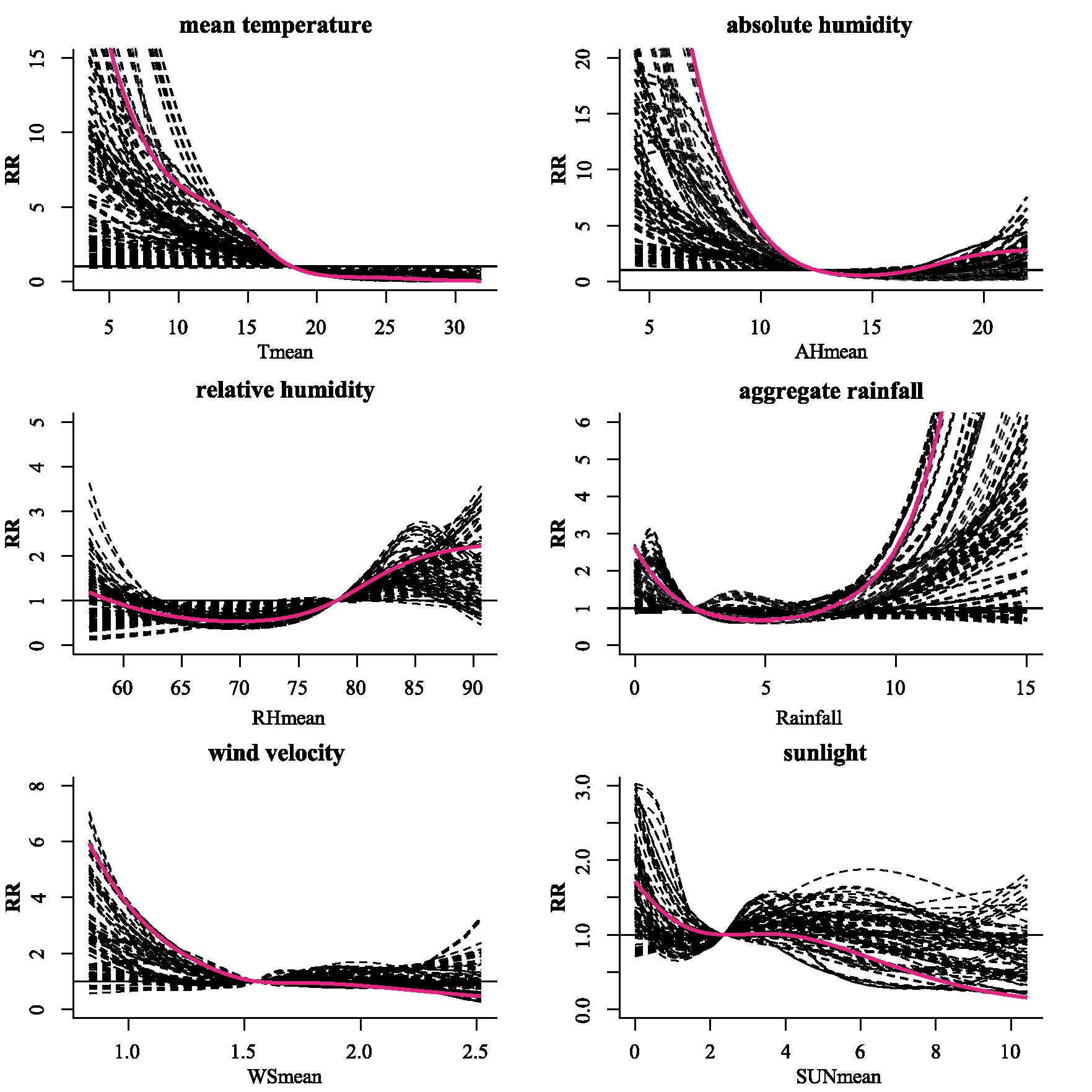

Supplement: S3 File — Each black line represents a combination and the red line stands the model established in this study. (TIF) [file pone.0246023.s003.tif]

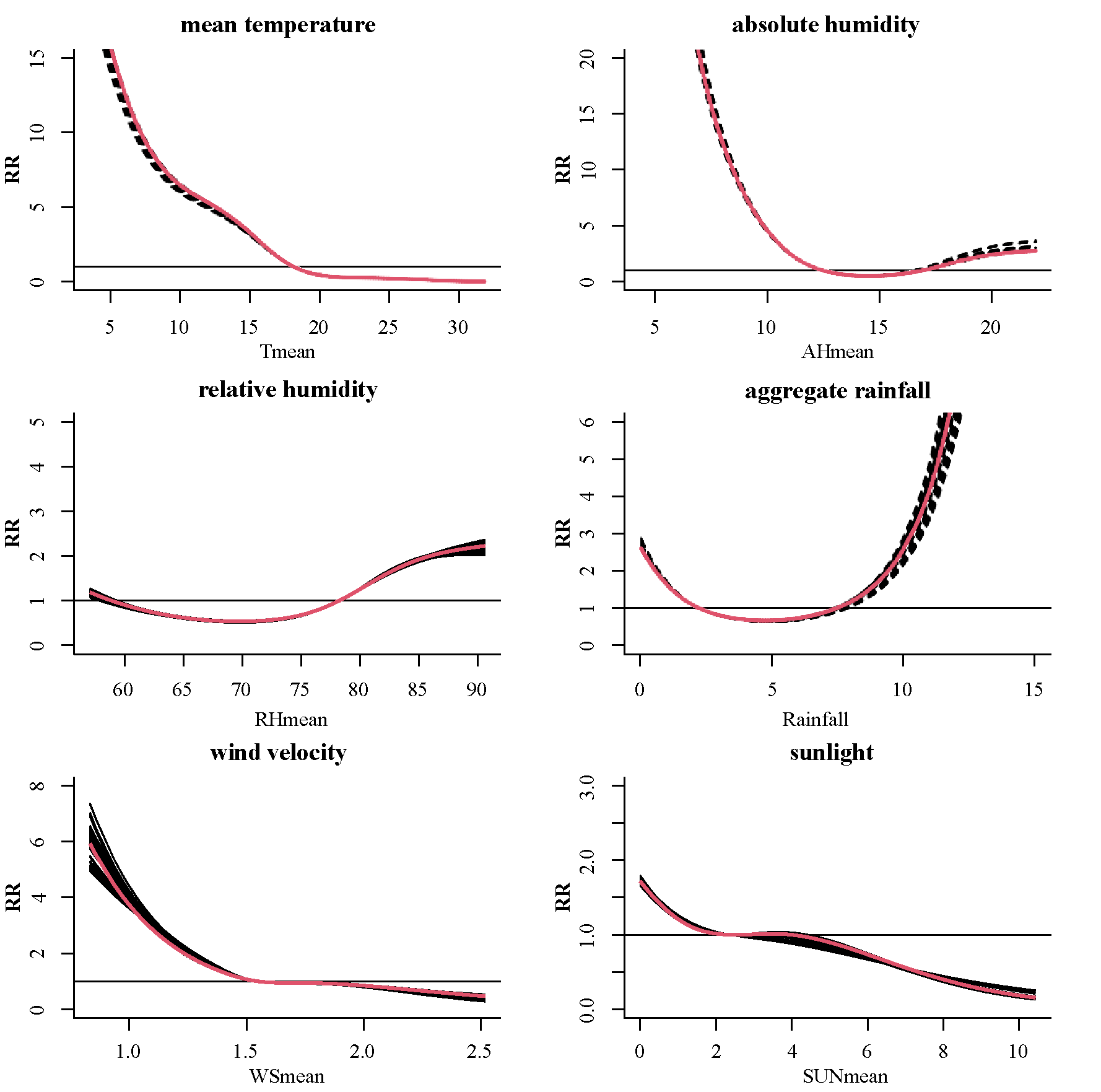

Supplement: S4 File — Each black line represents a combination and the red line stands the model established in this study. (TIF) [file pone.0246023.s004.tif]
